# A biomarker of aging, p16, predicts peripheral neuropathy in women receiving adjuvant taxanes for breast cancer

**DOI:** 10.1038/s41523-022-00473-3

**Published:** 2022-09-08

**Authors:** Natalia Mitin, Kirsten A. Nyrop, Susan L. Strum, Anne Knecht, Lisa A. Carey, Katherine E. Reeder-Hayes, E. Claire Dees, Trevor A. Jolly, Gretchen G. Kimmick, Meghan S. Karuturi, Raquel E. Reinbolt, JoEllen C. Speca, Erin A. O’Hare, Hyman B. Muss

**Affiliations:** 1 Sapere Bio, Research Triangle Park, NC USA; 2 School of Medicine, University of North Carolina at Chapel Hill, Chapel Hill, NC USA; 3 Lineberger Comprehensive Cancer Center, University of North Carolina at Chapel Hill, Chapel Hill, NC USA; 4 Duke University School of Medicine, Durham, NC USA; 5 MD Anderson Cancer Center, University of Texas, Houston, TX USA; 6 Ohio State University Comprehensive Cancer Center, Columbus, OH USA

**Keywords:** Cancer, Biomarkers

## Abstract

Identifying patients at higher risk of chemotherapy-induced peripheral neuropathy (CIPN) is a major unmet need given its high incidence, persistence, and detrimental effect on quality of life. We determined if the expression of *p16*, a biomarker of aging and cellular senescence, predicts CIPN in a prospective, multi-center study of 152 participants enrolled between 2014 and 2018. Any women with newly diagnosed Stage I–III breast cancer scheduled to receive taxane-containing chemotherapy was eligible. The primary outcome was development of grade 2 or higher CIPN during chemotherapy graded by the clinician before each chemotherapy cycle (NCI-CTCAE v5 criteria). We measured *p16* expression in peripheral blood T cells by qPCR before and at the end of chemotherapy. A multivariate model identified risk factors for CIPN and included taxane regimen type, p16Age Gap, a measure of discordance between chronological age and *p16* expression, and *p16* expression before chemotherapy. Participants with higher p16Age Gap—higher chronological age but lower *p16* expression prior to chemotherapy - were at the highest risk. In addition, higher levels of *p16* before treatment, regardless of patient age, conferred an increased risk of CIPN. Incidence of CIPN positively correlated with chemotherapy-induced increase in *p16* expression, with the largest increase seen in participants with the lowest *p16* expression before treatment. We have shown that *p16* expression levels before treatment can identify patients at high risk for taxane-induced CIPN. If confirmed, *p16* might help guide chemotherapy selection in early breast cancer.

## Introduction

Chemotherapy-induced peripheral neuropathy (CIPN) is among the most debilitating, common, and persistent chemotherapy toxicities^1–3^. CIPN can limit post-treatment quality of life, particularly important for patients receiving chemotherapy for stages I–III breast cancer^4,5^ since most receive a neurotoxic taxane and will have long-term survival. Unfortunately, up to 30% of these patients experience moderate to severe CIPN^6–8^. Symptoms occur predominantly in the hands and feet and include burning or shooting pain, paresthesia (numbness/tingling), pain perception abnormalities like allodynia and hyper- or hypo-algesia, temperature sensitivity, weakness, and, rarely, ataxia. In addition, multiple studies have shown that moderate to severe CIPN can be dose-limiting, raising concerns about compromised treatment efficacy^7,9,10^.

Even with dose-reductions, over 50% of patients receiving weekly paclitaxel experience limited recovery from CIPN^11^, with symptoms persisting five or more years post-treatment^3–5^ that dramatically impact quality of life^12–14^. Numbness in the feet can increase the risk of falling, particularly consequential in older patients where fractures can lead to inpatient rehabilitation and loss of independence^4,15–17^. Unfortunately, drug therapies to treat or prevent CIPN are largely ineffective^1,18^, especially among patients receiving taxanes^19^. Patients with severe, persistent, and painful CIPN may also be prescribed opioids, with a risk for opioid addiction^20–22^.

Given the potential severity of symptoms and lack of effective treatments, CIPN prevention becomes critical. Available strategies include cryotherapy^23–26^ and/or selection of a less neurotoxic agent. In Stages I–III breast cancer, neurotoxic taxanes (docetaxel and paclitaxel) are commonly used in both the adjuvant and neoadjuvant settings. Paclitaxel confers a much greater risk of CIPN than docetaxel^5,27^, therefore docetaxel may be preferred in at-risk patients. In addition, several preventive pharmacotherapies are under development^28^.

Identifying patients at-risk of CIPN is essential for prevention. Epidemiological risk factors include diabetes, obesity, and age^2,29–31^, though there is no consensus about their relative importance or application in CIPN prevention^2^. Recently, cellular senescence was found to positively associate with cisplatin-induced peripheral neuropathy in mice, and depletion of senescent cells abolished neuropathy^32^.

Cellular senescence is a fundamental mechanism of aging and plays a causative role in nearly all chronic age-related diseases and physical decline^32–39^. Senescent cells undergo permanent growth arrest, are resistant to apoptosis, and secrete both inflammatory and pro-fibrotic cytokines, disrupting tissue function and homeostasis^40,41^. Recent studies elegantly demonstrated that induction of senescence in just the immune compartment, and T cells specifically, can induce both senescence and organ damage in tissues throughout the body^42,43^. Expression of *p16*^*INK4a*^ (*p16*) mRNA in peripheral blood T lymphocytes has emerged as a key biomarker of senescence and a measure of senescent cell load^44^.

Cellular senescence has not been evaluated as a risk factor for peripheral neuropathy in humans. We hypothesized that *p16* expression would associate with the risk of CIPN.

## Results

### Participant demographic and clinical characteristics

Characteristics of 152 study participants with early-stage breast cancer receiving taxane-containing chemotherapies are shown in Table 1. Median age was 56 years (range of 24–83 years), 20% were black, 11% had diabetes, 53% received paclitaxel (48/81 weekly) and the remaining 47% received docetaxel. For analysis purposes, treatments were grouped based on the taxane type, as paclitaxel is more likely to induce CIPN than docetaxel^5,27^. Overall, 29% of participants experienced grade 2 or higher CIPN, of which 82% received paclitaxel and 18% docetaxel-based therapy. In a univariate analysis, none of the patient or clinical characteristics (except for chemotherapy regimen) were different between the CIPN and no CIPN groups, including age (*p* = 0.07, Student’s *t*-test), diabetes (*p* = 0.7, Chi-square test) and *p16* expression (*p* = 0.47, Student’s *t*-test).

**Table 1:** Demographic and clinical characteristics of the participants in this study.

Variable	All *N* = 152	Grade 2 + CIPN *n* = 44	No CIPN *n* = 108	*P*-value
**Age - median (SD)**	56 (13)	59 (11)	55 (13)	0.07
Range	24–83	34–83	24–77	
*p16*, log2 median (SD)	9.6 (0.9)	9.4 (0.9)	9.5 (0.9)	0.47
Race, *n* (%)				0.19
White	112 (74)	32 (73)	80 (75)	
Black	30 (20)	9 (20)	21 (20)	
Other	10 (6)	3 (7)	7 (5)	
Comorbidities, *n* (%)				
Diabetes	15 (11)	5 (12)	10 (10)	0.70
Peripheral vascular issues	3 (2)	1 (3)	2 (2)	1.00
Osteoporosis	15 (11)	3 (7)	12 (2)	0.55
Arthritis	45 (32)	17 (43)	28 (28)	0.09
High blood pressure	43 (30)	13 (32)	30 (30)	0.84
Coronary heart disease	5 (4)	0 (0)	5 (5)	0.32
Stroke	5 (4)	2 (5)	3 (3)	0.63
Liver or kidney disease	10 (7)	3 (8)	7 (7)	1.00
BMI—median (SD)	28.9 (6.3)	30.1 (6.6)	28.5 (6.2)	0.15
BMI ≥ 30, *n* (%)	59 (39)	19 (43)	40 (37)	0.48
Breast cancer stage, *n* (%)				0.15
I	32 (21)	5 (11)	27 (25)	
II	73 (48)	25 (57)	48 (44)	
III	44 (29)	13 (30)	31 (29)	
Missing data	3 (2)	1 (2)	2 (1)	
Hormone receptor and HER2 status, *n* (%)				0.86
ER+ or PR+ /HER2−	71 (47)	21 (48)	50 (46)	
HER2+	35 (23)	10 (23)	25 (23)	
ER−/PR−/HER2−	46 (30)	13 (30)	33 (31)	
Breast cancer surgery, *n* (%)				0.94
Lumpectomy	68 (45)	19 (43)	49 (45)	
Mastectomy	82 (54)	25 (57)	57 (53)	
None	2 (1)	0 (0)	2 (2)	
Adjuvant radiotherapy, *n* (%)	105 (72)	30 (68)	5 (74)	0.55
Chemotherapy regimen, *n* (%)				0.0001
Paclitaxel-containing	81 (53)	36 (82)	45 (42)	
Weekly paclitaxel	48 (32)	23 (52)	25 (23)	
Docetaxel-containing	71 (47)	8 (18)	63 (58)	
Regimen^1^				
AC-T	52 (34)	23 (52)	29 (27)	
AC-TC	17 (11)	5 (11)	12 (11)	
TC	41 (27)	2 (5)	39 (36)	
TC-H	25 (16)	5 (11)	20 (19)	
Other	17 (11)	9 (20)	8 (7)	
Anti-HER2 therapy, *n* (%)	35 (23)	10 (23)	25 (23)	1.00
Chemotherapy timing, *n* (%)				0.59
Neoadjuvant	64 (42)	20 (45)	44 (41)	
Adjuvant	88 (58)	24 (55)	64 (59)	
CIPN-CTCAE, *n* (%)				
Grade 0	53 (35)			
Grade 1	55 (36)			
Grade 2	43 (28)			
Grade 3	1 (1)			

^1^AC-T- doxorubicin, cyclophosphamide, paclitaxel; AC-TC- doxorubicin, cyclophosphamide, paclitaxel and carboplatin; TC- docetaxel and cyclophosphamide; TC-H docetaxel, carboplatin, and anti-HER2.

### Regression model of clinical variables and risk of CIPN

Since no variables in Table 1 were independent predictors of CIPN, we tested them in a multivariate regression analysis. Chronological age, race, and comorbidities were considered. We also included pairwise interactions, as variables like age, *p16* and comorbidities may not be independent (see Methods for details). Taxane type (paclitaxel vs docetaxel) was also included given the difference in CIPN incidence between these two agents. The optimal model from these variables (Model 1) is shown in Table 2. Taxane type, *p16* expression before chemotherapy, chronological age, arthritis, and osteoporosis, contributed to model performance.

**Table 2:** Performance and optimal variable combinations produced by multivariate regression analysis to predict risk of grade 2+ CIPN.

					Variable importance	
		Estimate	*p*	OR	Main Effect	Total Effect
**Model 1 variables**						
Taxane type [paclitaxel]		0.98	<0.0001			
*p16* pre-chemotherapy		−0.67	0.02			
Arthritis [yes]		0.62	0.03			
Age		−0.46	0.16			
Age*Osteoporosis [yes]		−0.49	0.14			
Osteoporosis [yes]		4.83	0.16			
Age*Arthritis [yes]		−0.03	0.23			
**Model 1 performance**						
AICc	150					
BIC	172					
Chi-square	34.9					
p value	0.0001					
Model 2 variables						
Taxane type [paclitaxel]		0.98	<0.0001	7.2 (2.9–17.5)	NA*	NA*
p16Age Gap		−0.047	0.01	0.95 (0.92–0.99)	0.406	0.519
*p16* pre-chemotherapy		1.22	0.04	3.4 (1.1–10.8)	0.363	0.477
**Model 2 performance**						
AICc	161					
BIC	173					
Chi-square	29.3					
p value	0.0001					

Variables tested in Model 1- taxane type, race, BMI ≥ 30, diabetes, peripheral circulatory issues, osteoporosis, arthritis, high blood pressure, liver or kidney disease, age, interaction of each comorbidity and age, and *p16* prior to chemotherapy.

Variables tested in Model 2- taxane type, race, BMI ≥ 30, diabetes, peripheral circulatory issues, osteoporosis, arthritis, high blood pressure, liver or kidney disease, age, interaction of each comorbidity and age, *p16* prior to chemotherapy, and p16Age Gap.

*Variable importance for categorical variables is not calculated.

### p16Age Gap

**Fig. 1:**
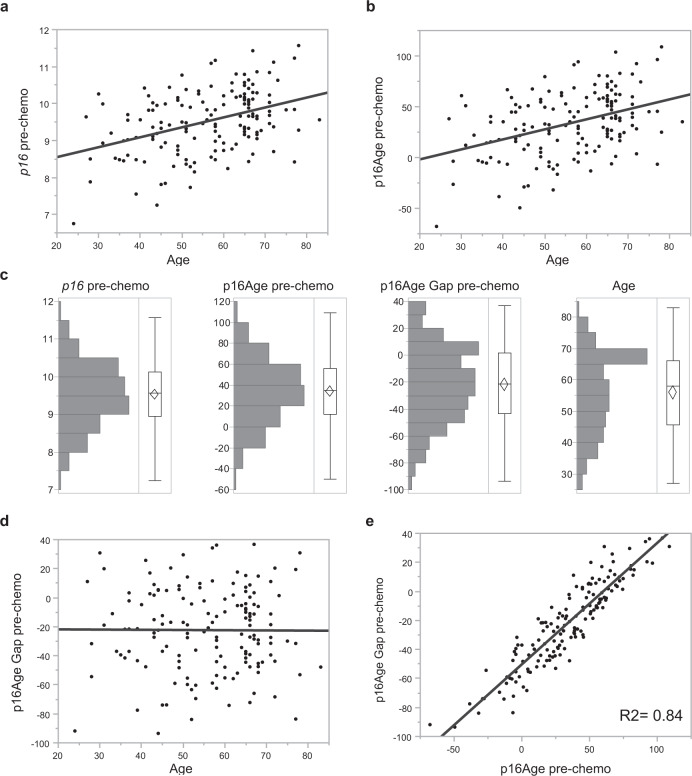
Expession of *p16* mRNA, p16Age, and p16Age Gap prior to chemotherapy. Correlation between chronological age and expression levels of *p16* mRNA (log2) (**a**), calculated p16Age (**b**) and p16Age Gap (**d**) and 16Age and p16Age gap (**e**). Distribution of *p16*, p16Age, p16Age Gap and chronological age are summarized in a histogram (**c**). The histogram shows a bar for grouped values of each continuous variable. The outlier box plot in each graph shows quantiles of continuous distributions. The horizontal line within the box represents the median sample value. The confidence diamond contains the mean and the upper and lower 95% of the mean. p16Age (and therefore log2 *p16*) is highly correlated with p16Age Gap.

Differences in the behavior of *p16* and age in the univariate vs. multivariate analyses prompted us to develop a measure called p16Age Gap, the difference between an individual patient’s pre-treatment *p16* and population-average *p16* levels by age. First, we developed a method to convert *p16* expression from log2 arbitrary units into years, and then directly compared *p16*-based age and chronological age (see Methods). Figure 1 shows a distribution of log2 *p16*, with *p16* converted to years (p16Age), and the difference between *p16* and chronological age (p16Age Gap). p16Age Gap can also be thought of as a residual in the *p16*/chronological age regression model. A negative p16Age Gap suggests that an individual has *p16* expression levels below an age-appropriate population mean; a p16Age Gap around zero suggests that *p16* expression is similar to the population mean; and positive p16Age Gap signifies *p16* expression above the population mean.

Distributions of *p16* (log2), p16Age, p16Age Gap, and chronological age in this study cohort are shown in Fig. 1c and are all normally distributed. Unlike p16Age, p16Age Gap does not correlate with chronological age (*p* = 0.94, Student’s *t*-test) (Fig. 1d), demonstrating that participants of all ages may exhibit age-inappropriate levels of *p16*. Larger absolute values of p16Age Gap values can represent younger participants who are molecularly older (positive p16Age Gap), or older participants who are molecularly younger (negative p16Age Gap). p16Age Gap is strongly associated with p16Age (R2 = 0.84; *p* < 0.0001, Student’s *t*-test; Fig. 1e), suggesting that variability in *p16* expression is the most important contributor to p16Age Gap, not differences in chronological age.

### P16Age Gap predicts risk of taxane-induced peripheral neuropathy

To assess the role of this new measure, p16Age Gap, as a predictor of CIPN risk, we built a second regression model (Table 2, Model 2) using p16Age Gap and the same variables tested in Model 1. The addition of p16Age Gap to these variables, which were used in building Model 1, reduced the number of variables to three, p16Age Gap, *p16*, and taxane shown to achieve similar performance to Model 1 (see Arc and BIC). When variables were analyzed for their individual contributions to the CIPN outcome (see Methods), p16Age Gap contributed 41% to the model as an individual predictor and 52% when considered with pre-chemotherapy *p16* expression. Patients with a negative p16Age Gap (chronologically older with lower *p16* expression) were at a higher risk for CIPN (OR 0.95, *p* = 0.01). Interestingly however, higher pre-chemotherapy *p16* expression was also associated with a higher risk of CIPN in a multivariate model (OR 3.4, *p* = 0.04).

**Fig. 2:**
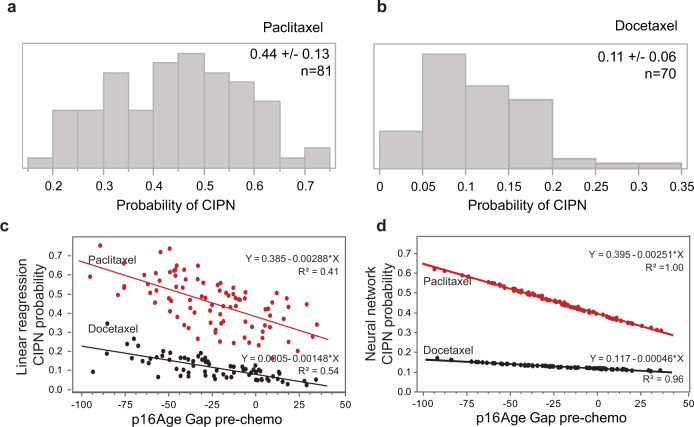
Correlation between p16Age Gap and probability of CIPN. Probability of developing grade 2+ CIPN, calculated from the linear regression Model 2 in participants receiving paclitaxel (**a**) or docetaxel (**b**) chemotherapy is shown in a histogram. Correlation between p16Age Gap single variable and a probability of CIPN derived from linear regression (**c**) or neural network (**d**) based model demonstrates significant contribution of p16Age Gap alone to the model.

The probability of CIPN in patients receiving paclitaxel- versus docetaxel-based chemotherapy was derived from regression Model 2 and shown in Fig. 2. Patients who received paclitaxel had mean probability of grade 2 or greater CIPN of 45% (CI 31–57%) (Fig. 2a). Patients who received docetaxel had a mean probability of grade 2 or greater CIPN of 11% (CI 5–17%) (Fig. 2b). Correlation between p16Age Gap and probability of CIPN for each taxane is shown in Fig. 2c. In patients receiving paclitaxel, the probability of CIPN increased from 14% for patients with p16Age Gap in lower quartile to 48% for patients in the upper quartile.

In addition to the regression model, we used the same variables to build a neural network to predict CIPN (see Methods). The relationship between the probability of CIPN and p16Age Gap as defined by the neural network algorithm is shown in Fig. 2d. Interestingly, while the neural network algorithm fit data better than linear regression analysis and produced a much higher R2 value, the slope coefficient for change in CIPN risk for paclitaxel patients was essentially identical between models (linear regression 0.00288, neural network 0.00251). This suggests that the linear regression model was sufficient to describe the relationship between p16Age Gap and CIPN incidence. Taken together, these findings suggest that systemic cellular senescence is an important risk factor for CIPN.

### Chemotherapy-induced increase in *p16* expression and taxane-induced peripheral neuropathy

**Fig. 3:**
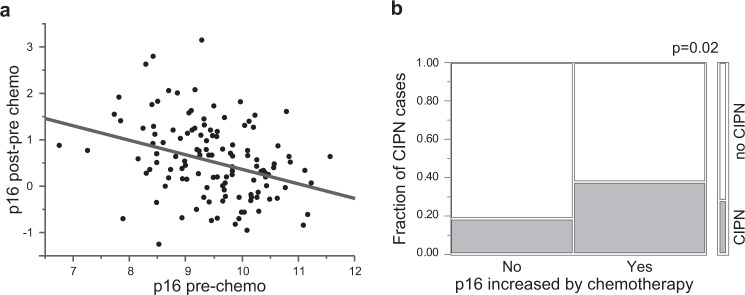
Chemotherapy-induced increase in *p16* as risk factor for CIPN. Participants with low *p16* were more likely to experience a chemotherapy-induced increase in *p16* and were more likely to develop taxane-induced CIPN. **a** Correlation between *p16* expression prior to chemotherapy and chemotherapy-induced change in *p16* expression measured at the end of chemotherapy regimen. **b** Mosaic plot of incidence of CIPN. CIPN group is shown in gray, no CIPN in white.

Finally, we asked why patients with lower baseline *p16* expression may be at higher risk of CIPN. We previously showed that *p16* expression prior to chemotherapy is inversely correlated with the magnitude of chemotherapy-induced *p16* increase^45^ (also Fig. 3a). Patients who experienced a post-chemotherapy increase in *p16* were twice as likely to develop CIPN (37.5% vs 18.3%, *p* = 0.02, Chi-square test) as patients whose *p16* did not change (Fig. 3b). Taken together, our results suggest that participants with lower *p16* expression prior to chemotherapy are more likely to have a larger chemotherapy-induced increase in *p16* expression and, more importantly, are more likely to experience grade 2 or higher CIPN.

## Discussion

Determining which patients are at risk of CIPN is a major unmet need in oncology, given its high incidence and persistence into survivorship. We examined if a measurement of cellular senescence, *p16* expression, is a risk factor for CIPN. We anticipated that higher *p16* would be associated with greater risk of CIPN as aging could be a risk factor^2^. Surprisingly, we found that patients who were chronologically older but molecularly younger (lower *p16* expression) as well as patients with high *p16* exression levels were at the highest risk for CIPN. Although our findings of higher CIPN risk for those with age-inappropriately low *p16* (negative p16Age Gap) initially seemed counterintuitive, we observed that patients with lower baseline *p16* expression are more likely to have a larger chemotherapy-induced increase in *p16*^45^ (Fig. 3a). And larger chemotherapy-induced change in *p16* expression (pre-treatment to post-treatment) is associated with highest CIPN risk (Fig. 3b).

One explanation for why a lower p16Age Gap value may confer higher risk of CIPN that we considered is that patients with a lower p16Age Gap maybe be percieved to be fitter by their oncologists and prescribed a more intense or longer treatment regimens. While total dose was not availbale in this dataset, addition of taxane regimen (weekly vs every 3 weeks) to Model 2 did not change model performance (Supplementary Table 1). p16Age Gap levels also did not differ between participants whose chemotherapy was discontinued or reduced due to toxicities (Supplementary Fig. 1), however a consideration of total dose delivered is planned for a follow-up study.

Peripheral neurotoxicity mechanisms of anticancer drugs are not fully understood and are likely to be complex. One hypothesis that could explain our findings centers on age-related changes in nerve conduction velocity. Because nerve conduction velocity is slower in older patients^46^, patients with a low senescent load may have better nerve conduction velocity before treatment than those with a higher p16Age Gap and experience a larger reduction in nerve conduction velocity than patients whose nerve conduction velocity is already low due to their age and age-appropriate *p16* (positive p16Age Gap). This reduction in velocity might be perceived by the brain as more severe CIPN. Nerve conduction velocity studies might verify this hypothesis but are beyond the scope of this study.

Presently, it is unclear why chemotherapy-induced *p16* expression is modulated by *p16* levels prior to chemotherapy. Tsygankov et al showed that *p16* expression plateaus in late middle age after increasing exponentially with age in early to mid-adulthood^47^. Thus, participants with lower baseline *p16* levels may have the capacity for a larger increase in *p16* following chemotherapy, while participants with higher baseline *p16* levels may have already reached a maximum threshold for senescent cell accumulation, suggesting that *p16* levels cannot increase further without causing morbidity. p16Age Gap, measured at baseline, can therefore be interpreted as a predictor of patients that will accumulate more senescent cells in response to chemotherapy, leading to higher *p16* expression and CIPN. Regardless of mechanism, our results, once validated, would allow identification of high risk patients, who could then consider CIPN prevention options like cryotherapy, substituting docetaxel for paclitaxel, or chemotherapy regimens without taxanes. Such high-risk patients would also be ideal candiates to be included in trials evaluating CIPN preventive strategies.

While our study provides evidence for a connection between p16, senescence, and CIPN in humans, a causal relationship between senescence and CIPN has been demonstarted in mice. Removal of the p16+ senescent cells in cisplatin-treated mice completely reversed symptoms of CIPN^32^. However, in this study *p16* expression was measured in dorsal root ganglia and not in blood. Although these discoveries may lead to ways to prevent CIPN through depletion of senescent cells (i.e. senolytic therapies), much work remains to understand human senescence and identify safe and efficacious senolytic therapies.

In this study, we did not find diabetes, obesity, or age to be important contributors to multivariate models of CIPN. A larger study is now underway (SENSE, NCT04932031) that will provide the opportunity to validate p16Age Gap findings, further interrogate comorbidities such as diabetes and age as risk factors, and analyze other variables not available in this study such as taxane dose intensity and metabolism. There is also great interest in genetic polymorhisms as predictors of CIPN with different chemotherapeutic agents, including taxanes, which may ulimately improve risk prediction^48^.

In addition to the sample size, another limitation of this study is that measurement of p16Age Gap was calculated using linear regression of *p16* versus age but, as noted above, the best fit between *p16* and chronological age is likely non-linear later in life. This may explain the extreme negative p16Age Gap values for some patients in our analysis. We are currenlty conducting a study to build a computational model of senescence in multiple patient cohorts to provide a better estimator of p16Age Gap. But regardless of the absolute value for the p16Age Gap, low *p16* expression in patients with higher chronologic age is a significant risk factor.

Our ongoing studies are designed to validate this p16Age Gap-based model for CIPN prediction for early-stage breast cancer patients and advance it into a lab-developed test to be used clinically to obtain a CIPN risk score. The planned studies will use patient reported outcomes (EORTC-QLQ-CIPN20) in addition to clinician-assessed (NCI-CTCAE) toxicity. If validated, this score may help to guide chemotherapy selection, given that regimens for this indication usually employ one of two different taxanes (paclitaxel or docetaxel) with similar efficacy but different risks of CIPN incidence^5,27^. Patients with age-inappropriate low *p16* may be offered docetaxel regimens, possibly in combination with other efforts to reduce CIPN such as cryotherapy, or closer monitoring for CIPN symptoms^23–26^. Additionally, anthracycline regimens that lack taxanes but have similar efficacy but different toxicity profiles, might be preferable for some pateints where even moderate risk of loss of function due to neuropathy may be unacceptable (for example musicians, surgeons, artists).

In summary, measures of senescent cell load could ultimately help guide clinicians to avoid dose-limiting toxicities and improve quality of life in breast cancer patients. These observations may also be clinically relevant in other cancers that are treated with neurotoxic chemotherapies.

## Methods

### Study participants

Women newly diagnosed with stage I–III breast cancer, enrolled in NCT02167932 or NCT02328313, who received a chemotherapy regimen containing a taxane^7^ and had *p16* mRNA expression measures were included in this analysis. All patients who were offered and consented to adjuvant or neoadjuvant chemotherapy were eligible to participate. Studies were led by the University of North Carolina with REX Healthcare, Ohio State University, MD Andersen, and Duke University participating, and were approved by the IRB of participating sites. The study was performed in agreement with the guidelines of the International Conference on Harmonization, the ethical principles in the Declaration of Helsinki, and all applicable regulations. All patients provided written informed consent before participation in any study-related activities.

### Chemotherapy regimens and measures

Chemotherapy regimens were classified as paclitaxel- or docetaxel-containing. CIPN toxicity data was collected as described^7^. For weekly regimens toxicity data was collected every other week so that all toxicity reports were either biweekly or triweekly. Briefly, CIPN symptoms were graded by an oncologist using the NCI-CTCAE v5 system. Symptoms were graded as none (0), mild (1), moderate (2), severe (3), or life-threatening (4). Clinicians assessed patients prior to each cycle of chemotherapy. In this study, measures to prevent CIPN such as cryotherapy or prescription medications were not captured as they were not widely utilized when the study was conducted.

### p16 expression

Peripheral blood samples were collected prior to starting chemotherapy and again at the end of chemotherapy, T cells were isolated, and *p16* mRNA expression was analyzed by real-time qPCR as described^49–51^, using reagents provided by Sapere Bio (SapereX). Positive and negative controls were included in each run; overall precision of *p16* measurement (biological and technical) was 0.8 Ct. Each measurement was performed once on each sample.

### p16Age Gap calculation

P16 expression levels were converted into equivalent years of aging (p16Age) using a linear regression formula derived from analysis of *p16* expression in 633 subjects^52^. The *p16* value corresponding to the participants’ chronological age at the start of chemotherapy was then subtracted from p16Age to calculate p16Age Gap.

### Statistical analysis

Chi-square tests, Fisher exact tests, and Student t tests were used to compare patient and clinical characteristics. All tests were two-sided with statistical significance set at 0.05. Analyses were conducted using SAS/JMP 15.1 software (SAS Institute Inc, Cary, North Carolina).

To generate a multivariate linear regression model of CIPN risk, chronological age, race, *p16* prior to chemotherapy, comorbidities, and their interactions were tested, as variables may not be independent. Comorbidities considered were obesity (BMI ≥ 30), diabetes, peripheral circulatory issues, osteoporosis, arthritis, high blood pressure, emphysema, and liver or kidney disease. Coronary heart disease and stroke were not used due to low prevalence in the CIPN group. Variables and their interactions were considered in the regression model and retained by forward stepwise addition to minimize the Akaike information criterion (AICc). The resulting CIPN probabilities were calculated and plotted. To determine the importance of each variable we calculated indices measuring the importance of factors in a model, in a manner independent of model type and fitting method. The fitted model is used only in calculating predicted values. This method estimates the variability in the predicted response based on a range of variation for each factor. If variation in the factor causes high variability in the response, then that effect is important to the model. Calculations assumed each variable was independent. In this analysis, for each factor, Monte Carlo samples were drawn from a uniform distribution defined by minimum and maximum observed values. Main Effect of importance reflects the relative contribution of that factor alone. Total Effect of importance reflects the relative contribution of that factor both alone and in combination with other factors. To mitigate overfitting when building a neural network model, 1/3 of the data set was reserved as a validation set using a holdback function and learning rate of 0.1. The model was built using a TanH activation function to fit one hidden layer with three nodes. The resulting probabilities of CIPN were calculated and plotted.

For the association between chemotherapy-induced change in *p16* and CIPN incidence, *p16* expression measured prior to chemotherapy was subtracted from p16 expression measured at the end of treatment and stratified into 2 groups: *p16* increase (change in *p16* > 0.4, half of assay precision) and no increase (*p16* change ≤ 0.4). Fisher’s exact test (2-sided) was used for group comparison.

### Reporting summary

Further information on research design is available in the Nature Research Reporting Summary linked to this article.

## Supplementary information

Supplementary Material

Reporting Summary

## Data Availability

Due to the informed consent and data privacy policies, the clinical data are not publicly available, i.e., accessible for anyone, for any purpose without a review by the UNC IRB on a project-by-project basis. Requests for raw data can be made to the corresponding author.
