# Supplementary material for: A biomarker of aging, p16, predicts peripheral neuropathy in women receiving adjuvant taxanes for breast cancer
Source: NPJ Breast Cancer. 2022 Sep 8;8:103. doi: 10.1038/s41523-022-00473-3 (PMC9458644; doi:10.1038/s41523-022-00473-3)
Supplement: Supplementary file 1 — Supplementary Material [file 41523_2022_473_MOESM1_ESM.pdf]

Supplementary Table 1. Addition of taxane administration frequency to the p16Age Gap-driven model does not change model performance.

**Variable**	**Estimate**	**p**
p16Age Gap	-0.047	0.01
p16 pre-chemo	1.25	0.04
Taxane type [paclitaxel]	0.88	0.002
Taxane frequency [weekly]	-0.24	0.32
**Model Performance**		
AICc	158	
BIC	172	
Chi-square	29.5	
p value	0.0001	

Supplementary Table 2. Dose reduction and discontinuation due to toxicities.

	**All**<br>**N=152**	**Grade 2+ CIPN**<br>**n=44**	**No CIPN**<br>**n=108**
Dose reduction, n (%)	46 (30)	18 (41)	28 (26)
Dose discontinuation, n (%)	22 (14)	10 (23)	12 (11)

Supplementary Figure 1

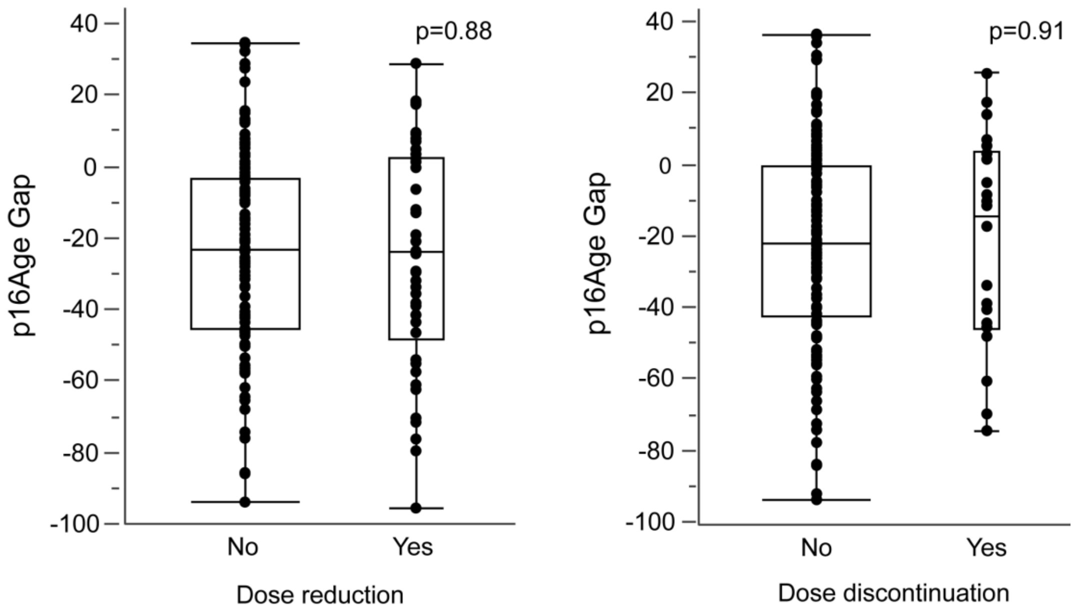

Supplementary Figure 1. P16Age Gap does not differ between participant groups whose taxane regimen was discontinued or reduced due to chemotherapy toxicity.
