# Supplementary material for: A biomarker of aging, p16, predicts peripheral neuropathy in women receiving adjuvant taxanes for breast cancer
Source: NPJ Breast Cancer. 2022 Sep 8;8:103. doi: 10.1038/s41523-022-00473-3 (PMC9458644; doi:10.1038/s41523-022-00473-3)
Supplement: Supplementary file 2 — Reporting Summary [file 41523_2022_473_MOESM2_ESM.pdf]

nature portfolio

Corresponding author(s): Hyman B. Muss

updated by author(s): 7.28.2022

Reporting Summary

Nature Portfolio wishes to improve the reproducibility of the work that we publish. This form provides structure for consistency and transparency in reporting. For further information on Nature Portfolio policies, see our Editorial Policies and the Editorial Policy Checklist.

Statistics

For all statistical analyses, confirm that the following items are present in the figure legend, table legend, main text, or Methods section.

n/a	Confirmed	
☐	☒	The exact sample size (*n*) for each experimental group/condition, given as a discrete number and unit of measurement
☐	☒	A statement on whether measurements were taken from distinct samples or whether the same sample was measured repeatedly
☐	☒	The statistical test(s) used AND whether they are one- or two-sided<br>*Only common tests should be described solely by name; describe more complex techniques in the Methods section.*
☐	☒	A description of all covariates tested
☐	☒	A description of any assumptions or corrections, such as tests of normality and adjustment for multiple comparisons
☐	☒	A full description of the statistical parameters including central tendency (e.g. means) or other basic estimates (e.g. regression coefficient) AND variation (e.g. standard deviation) or associated estimates of uncertainty (e.g. confidence intervals)
☒	☐	For null hypothesis testing, the test statistic (e.g. *F*, *t*, *r*) with confidence intervals, effect sizes, degrees of freedom and *P* value noted<br>*Give P values as exact values whenever suitable.*
☐	☒	For Bayesian analysis, information on the choice of priors and Markov chain Monte Carlo settings
☒	☐	For hierarchical and complex designs, identification of the appropriate level for tests and full reporting of outcomes
☒	☐	Estimates of effect sizes (e.g. Cohen's *d*, Pearson's *r*), indicating how they were calculated

Our web collection on statistics for biologists contains articles on many of the points above.

Software and code

Policy information about availability of computer code

Data collection	Date were collected in Redcap Data Base, HIPPA Compliant, UNC Chapel Hill, Chapel Hill NC. For details see: Harris, P. A., Taylor, R., Thielke, R., Payne, J., Gonzalez, N., & Conde, J. G. (2009). Research electronic data capture (REDCap)—a metadata-driven methodology and workflow process for providing translational research informatics support. Journal of Biomedical Informatics, 42(2), 377-381.
Data analysis	Analyses were conducted using SAS/JMP 15.1 software (SAS Institute Inc, Cary, North Carolina).

For manuscripts utilizing custom algorithms or software that are central to the research but not yet described in published literature, software must be made available to editors and reviewers. We strongly encourage code deposition in a community repository (e.g. GitHub). See the Nature Portfolio guidelines for submitting code & software for further information.

Data

Policy information about availability of data

All manuscripts must include a data availability statement. This statement should provide the following information, where applicable:

- Accession codes, unique identifiers, or web links for publicly available datasets
- A description of any restrictions on data availability
- For clinical datasets or third party data, please ensure that the statement adheres to our policy

Due to the informed consent form and data privacy policies, the clinical data is not publicly available, i.e., accessible for anyone, for any purpose without a review by the UNC IRB on a project-by-project basis. Requests for raw data can be made to the corresponding author.

Human research participants

Policy information about studies involving human research participants and Sex and Gender in Research.

Reporting on sex and gender	Women newly diagnosed with stage I-III breast cancer, enrolled in NCT02167932 or NCT02328313, who received a chemotherapy regimen containing a taxane and had p16 mRNA expression analysis were included in this analysis. All patients who were offered and consented to adjuvant or neoadjuvant chemotherapy were eligible to participate. Female sex was reported.
Population characteristics	See above
Recruitment	The patients had participated in a clinical trial that was previously described in a separate manuscript. No new data was abstracted for this study.
Ethics oversight	Study was approved by the IRB of participating sites as described in the manuscript.

Note that full information on the approval of the study protocol must also be provided in the manuscript.

Field-specific reporting

Please select the one below that is the best fit for your research. If you are not sure, read the appropriate sections before making your selection.

☒ Life sciences ☐ Behavioural & social sciences ☐ Ecological, evolutionary & environmental sciences

For a reference copy of the document with all sections, see nature.com/documents/nr-reporting-summary-flat.pdf

Life sciences study design

All studies must disclose on these points even when the disclosure is negative.

Sample size	Samples were collected from all patients that participated in a previously published clinical study. Patients who had both the biomarker of interest (p16 measurement prior to chemotherapy) and CIPN assessment by the clinician were included in the study cohort analysed in this manuscript as described in the Methods section.
Data exclusions	No data or samples were excluded
Replication	N/A
Randomization	N/A. Observational study design
Blinding	N/A

Reporting for specific materials, systems and methods

We require information from authors about some types of materials, experimental systems and methods used in many studies. Here, indicate whether each material, system or method listed is relevant to your study. If you are not sure if a list item applies to your research, read the appropriate section before selecting a response.

Materials & experimental systems

n/a	Involved in the study
☒	☐ Antibodies
☒	☐ Eukaryotic cell lines
☒	☐ Palaeontology and archaeology
☒	☐ Animals and other organisms
☐	☒ Clinical data
☒	☐ Dual use research of concern

Methods

n/a	Involved in the study
☒	☐ ChIP-seq
☒	☐ Flow cytometry
☒	☐ MRI-based neuroimaging

Clinical data

Policy information about clinical studies

All manuscripts should comply with the ICMJE guidelines for publication of clinical research and a completed CONSORT checklist must be included with all submissions.

Clinical trial registration	N/A. No randomization, observational study.
Study protocol	See above
Data collection	See above
Outcomes	See above
